# The Chinese thermal comfort dataset

**DOI:** 10.1038/s41597-023-02568-3

**Published:** 2023-09-28

**Authors:** Liu Yang, Shengkai Zhao, Yongchao Zhai, Siru Gao, Feixiang Wang, Zhiwei Lian, Lin Duanmu, Yufeng Zhang, Xiang Zhou, Bin Cao, Zhaojun Wang, Haiyan Yan, Hui Zhang, Edward Arens, Richard de Dear

**Affiliations:** 1 State Key Laboratory of Green Building in China, Xi’an University of Architecture and Technology, Yanta Road, 710055 Xi’an, Shaanxi China; 2 School of Architecture, Xi’an University of Architecture and Technology, Yanta Road, 710055 Xi’an, Shaanxi China; 3 Department of Architecture, School of Design, Shanghai Jiao Tong University, Dongchuan Road, 200240 Shanghai, China; 4 School of Civil Engineering, Dalian University of Technology, Linggong Road, 116024 Dalian, Liaoning China; 5 State Key Laboratory of Subtropical Building Science, Department of Architecture, South China University of Technology, Wushan Road, 510640 Guangzhou, Guangdong China; 6 College of Mechanical Engineering, Tongji University, Cao’an Road, 200092 Shanghai, China; 7 Department of Building Science, School of Architecture, Tsinghua University, Shuangqing Road, 100084 Beijing, China; 8 School of Architecture, Harbin Institute of Technology, Xidazhi Street, 150006 Harbin, Heilongjiang China; 9 School of Architectural and Artistic Design, Henan Polytechnic University, Jiefang Middle Road, 454002 Jiaozuo, Henan China; 10 Center for the Built Environment, University of California, Berkeley, 94720 Berkeley, CA USA; 11 School of Architecture, Design and Planning Wilkinson Building (G04-420), The University of Sydney, 2006 Sydney, New South Wales Australia

**Keywords:** Civil engineering, Psychology and behaviour

## Abstract

Heating and cooling in buildings accounts for over 20% of total energy consumption in China. Therefore, it is essential to understand the thermal requirements of building occupants when establishing building energy codes that would save energy while maintaining occupants’ thermal comfort. This paper introduces the Chinese thermal comfort dataset, established by seven participating institutions under the leadership of Xi’an University of Architecture and Technology. The dataset comprises 41,977 sets of data collected from 49 cities across five climate zones in China over the past two decades. The raw data underwent careful quality control procedure, including systematic organization, to ensure its reliability. Each dataset contains environmental parameters, occupants’ subjective responses, building information, and personal information. The dataset has been instrumental in the development of indoor thermal environment evaluation standards and energy codes in China. It can also have broader applications, such as contributing to the international thermal comfort dataset, modeling thermal comfort and adaptive behaviors, investigating regional differences in indoor thermal conditions, and examining occupants’ thermal comfort responses.

## Background & Summary

Climate change and sustainable human development are global challenges that are closely linked to the energy situation. Building energy consumption, particularly for heating and cooling purposes, constitutes a significant proportion of the overall energy demand^1,2^. Therefore, determining appropriate indoor thermal environmental parameters is crucial in reducing the energy use in buildings^3,4^. However, there are often large group and individual differences in building users’ responses in the same indoor environment^5,6^, and defining the comfort and health needs of building occupants requires access to a substantial amount of primary observational data. To facilitate international thermal comfort research, it is essential to establish a comprehensive and detailed field research thermal comfort dataset.

Over the years, researchers worldwide have conducted numerous thermal comfort field studies in various climate zones and building types, resulting in a wealth of thermal comfort data from building occupants under normal conditions. These data contribute to establishing a public data platform for thermal comfort research and a adaptative thermal comfort model, and provide basic parameters for the design of energy-saving buildings. At the same time, it improves the accuracy of building energy consumption prediction and plays a vital role in promoting the research frontier of thermal comfort. Currently, two major international platforms provide access to thermal comfort data: the SCATs^7^ database and the ASHRAE global thermal comfort database.

SCATs contain about 31,000 complete sets of paired subjective and objective comfort data from 26 buildings located in five European countries. It was established with funding from the European Union in the 1990s and provided the empirical data basis for the adaptive thermal comfort model in the European EN 15251^8^ (now EN 16798^9^) standard. The ASHRAE global thermal comfort database has two phases: database I and database II. Database I^10^ is a collection of 52 field studies conducted between 1982 and 1997 in 160 buildings worldwide, containing nearly 21,000 complete sets of paired subjective and objective comfort data, mostly from commercial office buildings. This database provided the empirical basis for the adaptive thermal comfort model^11,12^ in the ASHRAE Standard 55-2020^13^. The implementation of these standards marked the formal beginning of climatically adaptive thermal comfort building design and operational guidelines and the mainstream acceptance of the adaptive comfort theory. In 2018, the Center for the Built Environment at the University of California, Berkeley, and the Indoor Environmental Quality Laboratory at the University of Sydney established the thermal comfort database II^14^. It systematically collected raw data from the past two decades of thermal comfort research worldwide, following the template spelled out in the original database. This second-generation open-access repository of human thermal comfort in the built environment comprises approximately 100,000 sets of paired subjective and objective comfort data. While this database includes some Chinese field data, the sample size is relatively small (n = 8,235).

China is a vast country with several diverse climates, divided into five distinct zones from the thermal design of buildings points of view^15^, named as severe cold zone, cold zone, hot summer & cold winter zone, hot summer & warm winter zone, and mild zone. Different climate zones have different priorities and methods for building energy design, and occupants’ indoor thermal comfort needs should be met accordingly. A large amount of measured data is needed to accurately obtain the prediction model and design parameters to meet the indoor thermal comfort requirements. In recent years, the building science research community in China has conducted many field studies and accumulated results for different climate zones, building types and populations^16–20^. However, China’s current indoor thermal environment standard either directly follow foreign standard, such as GB/T 5701-2008^21^, GB/T 18049-2017^22^, or lack sufficient basic data support, such as GB/T 50785-2012^23^, GB 50736-2012^24^. To date, there is no indoor thermal environment design parameter system that reflects the actual characteristics of China or the thermal comfort characteristics of Chinese people. Additionally, there is no interior design parameter standard with China’s independent intellectual property rights and a unified database of indoor thermal environment parameters, hindering effective support of building energy-saving design.

During “the 13th Five-Year” Plan period, a project launched which was based on the National Key R&D Program. Its main objective was to address the existing problems of insufficient coverage of standardized parameters for building energy-saving design, non-standardized acquisition and processing methods, and failure to meet the demand for refined design. One of the project’s tasks was to establish a comfort field study dataset that could serve the needs of building energy efficiency projects. To achieve this goal, we accumulated field data over the past 20 years that covered typical climate characteristics, building thermal performance, indoor thermal environment of HVAC types, and subjective response characteristics of personnel in China. The dataset includes around 42,000 sets of data from different climate zones, seasons, building types, and HAVC end forms. The original data underwent standardization through data grading and quality control, and the thermal comfort dataset based on the thermal adaptation characteristics of the Chinese population was established. This dataset serves as a standardized indoor database aimed at meeting the design needs of building energy efficiency projects.

The Chinese thermal comfort dataset is a valuable resource that contains standardized measurement data, including indoor physical environments and subjective thermal comfort evaluations. Since its creation in 2018, it has yielded significant research results^25–28^. In this paper, we provide a comprehensive procedure for the standardized collection, cleaning, processing, and consolidation of data into the dataset. Further improvements and expansions to the Chinese thermal comfort dataset would provide essential data support for the development of reliable and accurate predictive models for adaptive thermal comfort and building energy efficiency design parameters.

The global thermal comfort research community can use the open-access Chinese thermal comfort dataset to:

- Analyze the indoor thermal environment parameters and their distribution in diverse building types across different climate zones in China.
- Investigate the thermal adaptation behaviors of building occupants in various Chinese climate zones.
- Collect building interior design parameters that meet the thermal comfort needs of people in different Chinese climate zones.
- Develop an adaptive thermal comfort model based on empirical field data collected exclusively in China, tailored to the specific characteristics of the Chinese population and their indoor thermal environment.

## Methods

**Fig. 1:**
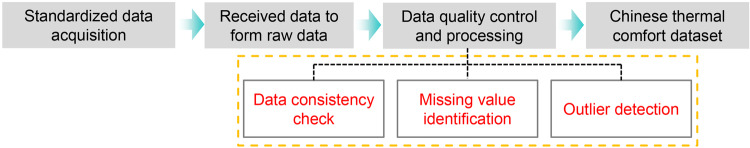
Flowchart of the data collection and quality control processes.

The Chinese thermal comfort dataset resulted from a collaborative effort by seven universities in China with the goal of establishing a unified and standardized dataset. After rigorous quality control and data processing (as shown in Fig. 1), the dataset contains approximately 42,000 sets of data. In the Method section, we provide an overview of the data collection process, as well as the processing and structure of the dataset.

### Data collection

**Fig. 2:**
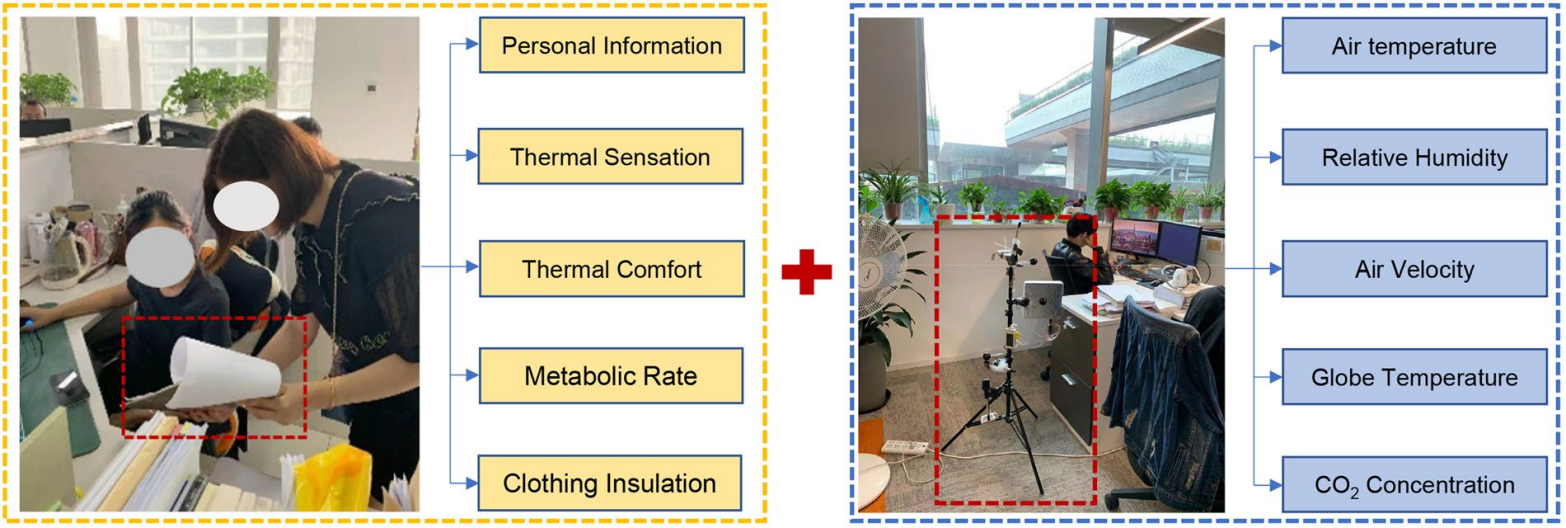
Subjective questionnaire and physical environment measurements.

Members of the Chinese thermal comfort research community located in China provided their original thermal comfort field study data. To ensure accuracy and reliability, each member adopted standardized data acquisition methods. Indoor thermal environment parameters test methods and test instrument accuracy according to the requirements of JGJ/T 347-2014^29^ and ISO 7726-1998^30^ standards. Subjective evaluation questionnaire followed ASHRAE 55-2020^13^, ISO 10551-2019^31^, GB/T 50785-2012^23^, GB/T 18977-2003^32^ standard and were structured into a unified data template. Figure 2 shows the subjective questionnaire evaluation and physical environment parameter test scenarios. Table 1 presents the institutes that contributed to each dataset and the corresponding amount of data. Generally, the data for each climate zone were provided by institutes located in that specific zone. Outdoor weather data were derived from surface meteorological stations across China acquired from the National Meteorological Information Center, China Meteorological Administration (http://data.cma.cn).

**Table 1:** Data contributor in thermal comfort dataset of China.

Institutions	Climate zone	Building type	Number	Publications
Harbin Institute of Technology (HIT)	SC	Office, Dormitory, Residential, Educational	Class I: 1318 Class II: 1612 Class III: 2206	Wang *et al*.^44–46^ Su *et al*.^28,39^ Du *et al*.^25–27^
Tsinghua University (QHU)	C, M	Office, Dormitory, Educational	Class I: 617 Class II: 2041 Class III: 656	Cao *et al*.^41,47,48^ Jia *et al*.^49^ Mou *et al*.^50^ Luo *et al*.^16,51^
Xi’an University of Architecture and Technology (XAUAT)	SC, C, HSCW, HSWW, M	Office, Residential	Class I: 1879 Class II: 7839 Class III: 0	Yang *et al*.^17,36^ Yan *et al*.^35,52–54^
Dalian University of Technology (DLUT)	SC, C, HSCW	Office, Dormitory, Residential, Others	Class I: 746 Class II: 1742 Class III: 738	Sun *et al*.^55^ Du *et al*.^25–27^ Su *et al*.^28^
Shanghai Jiao Tong University (SJTU)	HSCW, HSWW	Office, Dormitory, Residential, Educational	Class I: 605 Class II: 2384 Class III: 3628	Xu *et al*.^56^ Du *et al*.^25–27^
Tongji University (TJU)	SC, C, HSCW, HSWW, M	Office, Dormitory, Residential, Educational	Class I: 2081 Class II: 2128 Class III: 1319	Zhou *et al*.^57,58^
South China University of Technology (SCUT)	HSWW	Office, Dormitory, Residential	Class I: 0 Class II: 8438 Class III: 0	Zhang *et al*.^20,38,59^

**Fig. 3:**
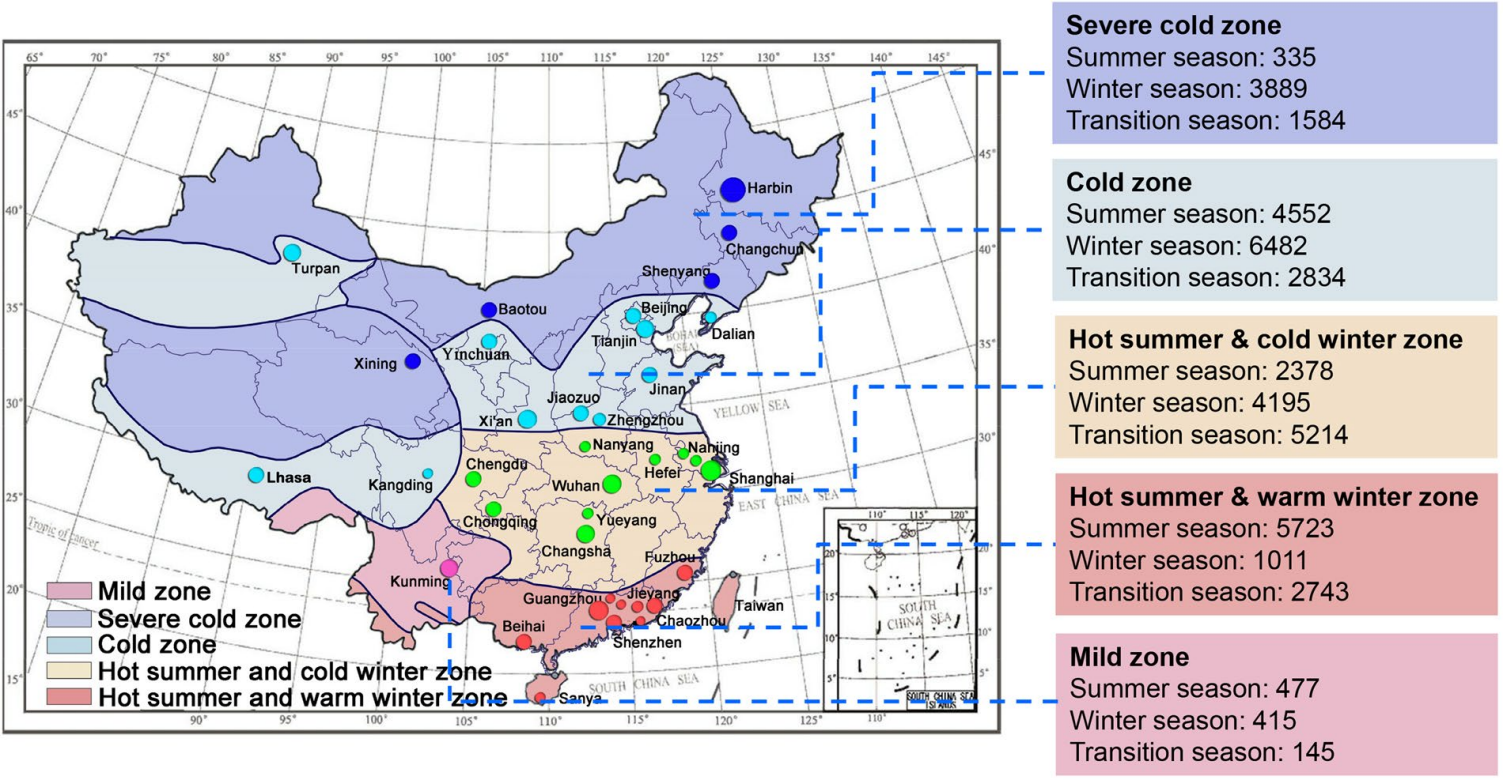
Location of the field studies contained in thermal comfort dataset of China. The summer moths are June, July, and August. The transitional seasons include spring (March, April, and May) and autumn (September, October, and November). The winter months are December, January, and February.

Figure 3 displays the distribution of research locations across the country, with cities having a sample size greater than 100 located on the map. The maps use five different color blocks represent different climate zones. It can be observed that except for the mild zone, all other climate zones have data from more than five typical representative cities contributing research data to the database. According to the unified indoor data acquisition method, each research community members divided the work in residential, dormitory, office, and classroom buildings in five climate zones. After data quality control, the final database contains a total of 41,977 sets of data, including 5,808 groups in severe cold zones, 13,868 groups in cold zones, 11,787 groups in hot summer & cold winter zones, 9,477 groups in hot summer & warm winter zones, and 1,037 groups in mild zones.

### Data grading

To address the various requirements for indoor thermal environments in building energy conservation designs, the Chinese thermal comfort dataset has established three levels of data classification based on the actual needs of building energy conservation design. These include indoor thermal environment parameters obtained directly from the original study data, subjective evaluations at all levels, and indoor thermal environment derived parameters obtained through calculation (such as mean radiant temperature). To ensure consistency in data naming, data type, and format, each dataset received from contributors was graded according to a three-level grading standard and filled into the corresponding Excel template table. The dataset is categorized into three levels (as detailed in Table 2) based on the information content of the data and covers five climatic regions in China, totaling 41,977 data sets. This includes 7,246 sets of Class I data, 26,184 sets of Class II data, and 8,547 sets of Class III data, as presented in Table 3.

**Table 2:** Classification of the indoor environment parameters in the Chinese thermal comfort dataset.

Class	Basic parameters	Calculated parameters	Other parameters	Instrument equipment
I	Air temperature*, Relative humidity*, Air velocity*, Globe temperature*, Average temperature of each surface of the room	Mean radiant temperature, Radiant temperature asymmetry	Clothing insulation, Metabolic rate and other personnel parameters, Subjective evaluation, Building space dimensions and personnel location, Building type, HVAC equipment type, Test data, Climate zone	Air temperature^#^: −10–50 °C (range), ±0.5 °C (required), ±0.2 °C (desirable); Relative humidity: 0–100% (range), ±5% (required), ±2.5% (desirable); Air velocity: 0–10 m/s (range), ±0.05 m/s (required), ±0.02 m/s (desirable); Globe temperature: −10–50 °C (range), ±0.5 °C (required), ±0.2 °C (desirable); Surface temperature: −10–50 °C (range), ±1 °C (required), ±0.2 °C (desirable).
II	Air temperature, Relative humidity, Air velocity, Globe temperature	Mean radiant temperature	Clothing insulation, Metabolic rate and other personnel parameters, Subjective evaluation, Building type, HVAC equipment type, Outdoor environmental parameters, Test data, Climate zone	
III	Air temperature, Relative humidity	/	Clothing insulation, Metabolic rate and other personnel parameters, Subjective evaluation, Building type, HVAC equipment type, Test data, Climate zone	

*Three measurement height, sitting position is 0.1 m, 0.6 m, 1.1 m, standing positions is 0.1 m, 1.1 m, 1.7 m; ^#^Respectively are measuring parameter, measuring range and measuring accuracy.

**Table 3:** Thermal comfort standardization dataset.

Climate zone	Sample size			Total sample size
	Class I	Class II	Class III	
Severe cold zone	1318	2250	2240	5808
Cold zone	4019	8619	1230	13868
Hot summer & cold winter zone	1909	5026	4852	11787
Hot summer & warm winter zone	/	9463	14	9477
Mild zone	/	826	211	1037
Total	7246	26184	8547	41977

### Data pre-processing

To ensure the reliability and rationality of building standards, it is essential to maintain high-quality data in the dataset. Therefore, all data provided by contributors undergo strict quality control before incorporation into the dataset. For indoor temperature, relative humidity, air velocity, mean radiation temperature, clothing insulation, metabolic rate, and subjective evaluation data collected, a standardized processing method was established to ensure the accuracy and reliability of the original data. This includes developing a standardized processing method for indoor temperature, relative humidity, air velocity, mean radiation temperature, clothing insulation, metabolic rate, TSV (thermal sensation vote), TCV (thermal comfort vote), TAV (thermal acceptability vote), and other original data collected in the dataset.

Before missing values are processed, the data is pre-processed to make it consistent and easy to process. This included:

- Data simplification. To facilitate the processing of the data, an excel sheet was used to separate numbers from Chinese characters (such as 0 (neutral)), letters and Chinese characters (such as A. male, B. female), or Chinese characters from Chinese characters (such as underwear┊T-shirt┊autumn clothes┊autumn pants) in the original data.
- Questionnaire integration. Using MATLAB programming method to realize the subjective data collation. The thermal sensation scale is based on the ASHRAE Standard 55-2020^13^ 7-point voting scale, with -3 (cold), -2 (cool), -1 (slightly cool), 0 (neutral), 1 (slightly warm), 2 (warm), 3 (hot). The thermal comfort scale is 6 - point scale with 0 (very comfortable), 1 (comfortable), 2 (just comfortable), 3 (just uncomfortable), 4 (uncomfortable), 5 (very uncomfortable). The thermal acceptability scale is 4-point scale with -1 (unacceptable), -0.01 (just unacceptable), 0.01 (just acceptable), 1 (acceptable).
- Metabolic rate. The dataset includes metabolic rate values of Chinese population under different activity states, which were tested by Zhai *et al*.^33^ using indirect calorimetry. The activity statuses of the subjects when they filled out the questionnaire were collected and converted into metabolic rate values. Sitting metabolic rate was 0.9 met, sitting typing was 1.0 met, sitting document filing was 1.2 met, standing office was 1.1 met, standing document filing was 1.3 met, and walking at 2 km/h was 2.1 met.
- Clothing insulation. Subjects select the corresponding clothing type based on their current clothing when filling in the questionnaire. If the clothing type was not listed in the questionnaire, they could choose a similar clothing type. The clothing insulation of a single garment was assigned according to the ASHRAE 55-2020^13^, while the insulation value of a suit was calculated by adding and summing the insulation values of each garment.

### Database structure

**Fig. 4:**
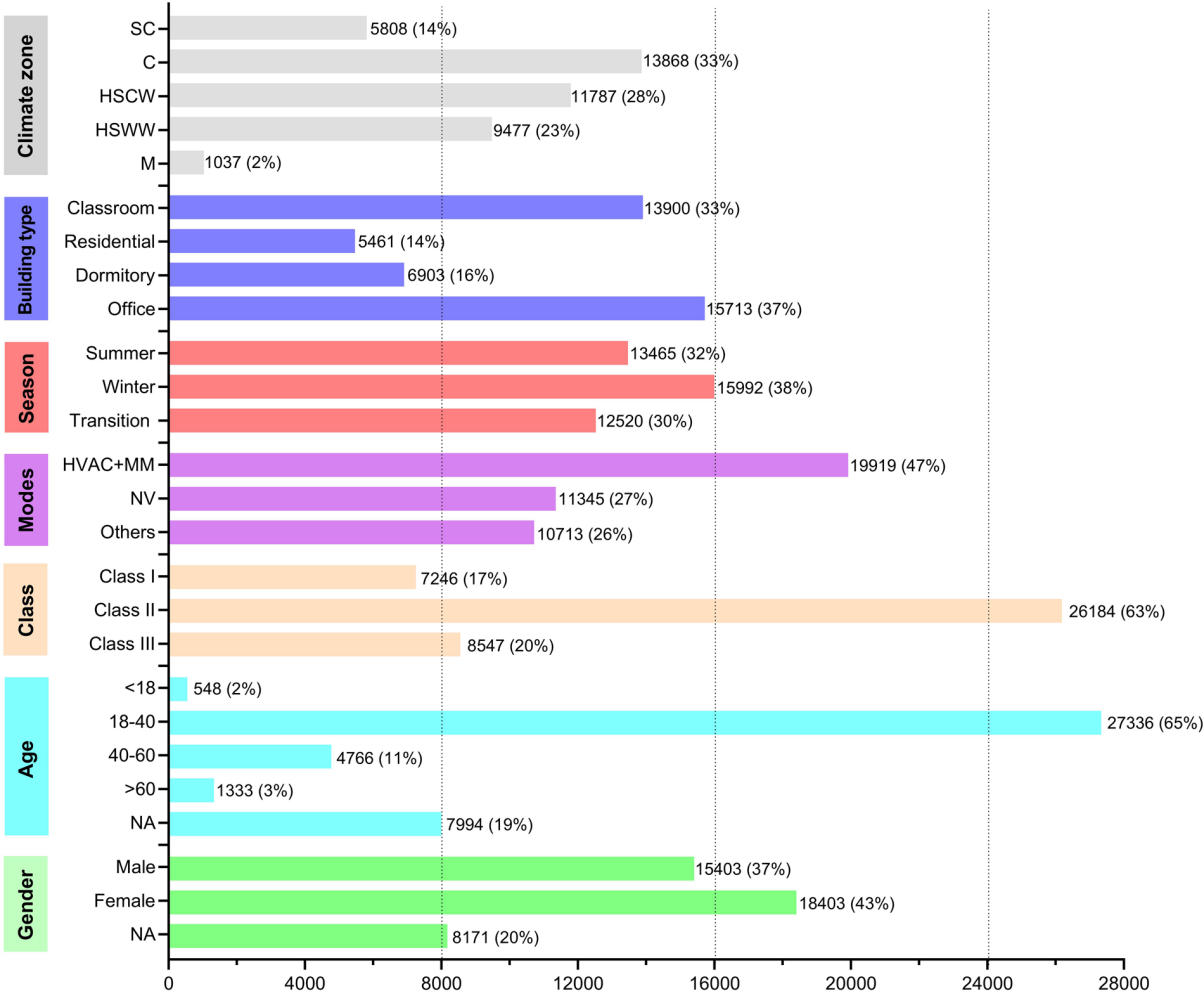
Distributions of thermal comfort dataset under different conditions.

The research team created the dataset using a standardized spreadsheet format. The dataset structure contains seven main categories: basic identifiers, building information, subject’s personal information, subjective thermal comfort information, indoor physical parameters, indoor calculated parameters, and outdoor environment parameters. To facilitate efficient data location and processing, each row of data in the tables is numbered. The data number consists of three parts: school code (abbreviation of each data providing unit), data level (1st, 2nd, and 3rd for Class I, Class II, and Class III, respectively), and data serial number (four Arabic digits). For example, XAUAT1st2400. Tables 4, 5 presents the complete list of variables in the dataset and their coding conventions. Figure 4 displays the distributions of thermal comfort data under different conditions.

**Table 4:** Variable coding conventions, including basic identifiers, building information, subject’s personal information, and subjective thermal comfort information.

Type of data	Description
**A. Basic Identifiers**	
A1. Code	Unit-Class-Number (For example: XAUAT-1st-0001)
A2. Date	Year-Month-Day-Hour (YYYY-MM-DD-HH)
A3. Data Contributor	Provide data researcher
A4. Season	Summer Season, Winter Season, and Transition Season
A5. City	City of the study was done
A6. Climate Zone	Severe Cold Zone (SC), Cold Zone (C), Hot Summer & Cold Winter Zone (HSCW), Hot Summer & Warm Winter Zone (HSWW), Mild Zone (M)
**B. Building Information**	
B1. Building Type	Office, Dormitory, Residential, Educational, Others
B2. Building Function	(Bedroom, Living Room, Office, Laboratory….)
B3. Floors	The floor where the research room is located
B4. Building Operation Mode	HVAC (including Radiant System and Convective System), Mixed Mode, Naturally Ventilated, Others
B5. Room Size	The length, width, and height of the room (m)
**C. Subject’s Personal Information**	
C1. Sex	Male, Female, Undefined
C2. Age	Age of the subjects
C3. Height	Height of the subjects (cm)
C4. Wight	Weight of the subjects (kg)
C5. Living Years	The number of years of the subjects lived in the survey city
**D. Subjective Thermal Comfort Information**	
D1. Thermal Sensation Vote	ASHRAE thermal sensation vote, from -3 (cold) to +3 (hot)
D2. Thermal Comfort Vote	Very comfortable (0), Comfortable (1), Just comfortable (2), Just uncomfortable (3), Uncomfortable (4), Very uncomfortable (5)
D3. Thermal Acceptability Vote	Unacceptable (-1), Just unacceptable (-0.01), Just acceptable (0.01), acceptable (1)
D4. Thermal Preference	Cooler, No change, Warmer
D5. Clothing Insulation	Intrinsic clothing ensemble insulation of the subjects (clo)
D6. Metabolic Rate	Activity status at the time of the survey (met), refer to ASHRAE 55-2020^13^

**Table 5:** Variable coding conventions, including indoor physical parameters, indoor calculated parameters, and outdoor environment parameters.

Type of data	Description
**E. Indoor Physical Parameters**	
E1. Indoor Air Temperature (T_a_)	Air temperature measured in the occupied zone at 0.1, 0.6, and 1.1 m above the floor (°C)
E2. Indoor Relative Humidity (RH)	Relative humidity measured in the occupied zone at 0.1, 0.6, and 1.1 m above the floor (%)
E3. Indoor Air Velocity (V)	Air velocity measured in the occupied zone at 0.1, 0.6, and 1.1 m above the floor (m/s)
E4. Globe Temperature (T_g_)	Globe temperature measured in the occupied zone at 0.1, 0.6, and 1.1 m above the floor (°C)
E5. Room Surface Temperature	Measured internal surface temperature of floor, wall, and roof surface (°C)
**F. Indoor Calculated Parameters**	
F1. Operative Temperature (T_op_)	Calculated operative temperature in the occupied zone (°C), it was calculated according to ASHRAE 55-2020^13^: T_op_ = A × T_a_ + (1-A) × T_r_, when v < 0.2 m/s, A = 0.5; 0.2 < v < 0.6 m/s, A = 0.6; 0.6 < v < 1.0 m/s, A = 0.7
F2. Mean Radiant Temperature (T_r_)	Calculated mean radiant temperature in the occupied zone (°C), it was calculated according to ISO 7726-1998^30^: T_r_ = [(T_g_ + 273)^4^ + 2.5 × 10^8^ × v^0.6^ (T_g_-T_a_)]^1/4^-273
F3. Radiant Temperature Asymmetry	Calculated radiant temperature asymmetry in the occupied zone (°C)
F4. PMV	Predicted mean vote, it was calculated according to ASHRAE 55-2020^13^
F5. PPD	Predicted percentage of dissatisfied, it was calculated according to ASHRAE 55-2020^13^
**G. Outdoor Environment Parameters**	
G1. Real-time Outdoor Temperature	Real-time outdoor temperature when the field study was done (°C)
G2. Mean Daily Outdoor Temperature	Daily mean outdoor temperature when the field study was done (°C)
G3. Monthly Mean Outdoor Temperature	Monthly mean outdoor temperature when the field study was done (°C)
**G4. 7 or 15-Day Running Mean**	
Outdoor Temperature (T_rm-7_ or T_rm-15_)	7 or 15 - day running mean outdoor temperature when the field study was done (°C), it was calculated according to ASHRAE 55-2020^13^: T_rm-7/15_ = (1-α)[t_e(d-1)_ + αt_e(d-2)_+…], α = 0.8
G5. Mean Daily Outdoor Relative Humidity	Daily mean outdoor relative humidity when the field study was done (%)
G6. Mean Daily Outdoor Air Velocity	Daily mean outdoor air velocity when the field study was done (m/s)

Table 4. Variable coding conventions, including basic identifiers, building information, subject’s personal information, and subjective thermal comfort information.

#### Ethics and consent

All data contained within this dataset were obtained through thermal comfort field surveys with strict adherence to ethical standards regarding human subject research. The survey instrument included questions regarding basic participant information (e.g., age, height, weight, gender, clothing style, and active state) as well as subjective evaluations of the indoor thermal environment. Surveys conducted from 2001 to 2016 were voluntary and informed consent was obtained from all respondents. Participants were fully informed about the content and purpose of the study, and the surveys were designed and conducted to avoid any invasion of personal privacy or harm to participants’ well-being.

For surveys administered from 2017 to 2021, approval was obtained from the Committee for Protection of Human Subjects at the University of California, Berkeley (CPHS Protocol Number: 2011-04-3163). This dataset and all accompanying survey data will be included in the future international thermal comfort Database II^14^. Prior to each field study, the informed consent of each interviewee will be obtained and it is up to the interviewee to decide whether to cooperate with the survey. Participants have the right to withdraw from the survey at any time or not to answer individual questions.

We respect the autonomous decisions of all subjects regarding their participation. All participants’ personal information will remain confidential and anonymous in any resulting publications or databases.

## Data Record

The data records listed in this section are available from the OSF website for public use^34^. Users can access and download the data without creating a platform account according to their personal needs. The data recording primarily comprises three aspects: data templates for data collection, the questionnaire, and the final dataset.

### Data templates for data collection

File format: Microsoft Excel (.csv). The files contain data templates that investigators use for data collection.

### Questionnaires

File format: Adobe pdf (.pdf). These documents consist of three parts: indoor physical environment parameter record form, subjective questionnaire (excepting the clothing insulation section), and the clothing insulation questionnaire.

- Physical environment parameter: It is used for staff to record indoor physical environment parameters.
- Subjective questionnaire: It is used to record basic personal information of subjects and subjective evaluation of indoor thermal environment of subjects.
- Clothing insulation: It is used to record the dressing condition of the subject.

### All datasets

File format: Microsoft Excel (.csv) or SQL. The dataset is divided into three files, named as Chinese Thermal Comfort Dataset Class I, Chinese Thermal Comfort Dataset Class II, and Chinese Thermal Comfort Dataset Class III.

### Program

File format: Adobe pdf (.pdf). The name of program is building indoor thermal environment data processing system. These files contain content for processing the raw datasets and technical validation.

## Technical Validation

The on-site thermal comfort survey data, obtained through a combination of field tests and survey questionnaires, may be affected by data quality issues resulting from instrument failure and errors made during data input by personnel. To ensure the dataset’s reliability and accuracy, six factors affecting human thermal comfort and subjective evaluation indexes were subject to quality control measures such as missing value identification, data consistency check, and outlier detection.

### Missing value identification

Two main types of missing data exist: 1) when a certain data point is absent, and 2) when the data point is indicated as “NA” or “0”. If missing data is due to the personnel negligence, the original data can be returned to the original contributors to check and confirm the missing data and refill it when available. If the missing data cannot be retrieved, the data will be downgraded based on the data grading principle, and data that doesn’t correspond to the corresponding level will be eliminated.

### Data consistency check

To ensure data consistency, it is essential to consider factors such as environmental conditions, occupants’ habits, and indoor data acquisition characteristics. The following are the reasonable ranges for the different variables:

- Indoor air temperature. Based on existing studies^35^, unheated rooms in winter should not have indoor temperature below 0 °C, while rooms with heating equipment should not be below 10 °C. For summer, in Turpan, a typical hot and dry climate area in China, the indoor temperature should not exceed 45 °C^36^, and for other survey sites, it should not be higher than 35 °C.
- Relative humidity. Existing studies have indicated that relative humidity is generally not lower than 5%^37^. In hot-humid climate region in China, such as Guangzhou and Shenzhen, the humid climate cause indoor condensation, leading to indoor relative humidity levels of up to 100%^38^. Therefore, a reasonable range for indoor relative humidity is considered to be from 5% to 100%.
- Globe temperature. In real environmental buildings, there may be radiation near the exterior wall or window^39^, but people typically reduce discomfort by using curtains and other behavioral adjustment. Therefore, the reasonable range of globe temperature is consistent with indoor temperature.
- Air velocity. In building with naturally ventilated, indoor air velocity is generally relatively high. Based on field investigation results^40^, the maximum air velocity should not exceed 3 m/s. When doors and windows are closed, indoor air velocity is low, and air velocity is close to 0 m/s. Therefore, a reasonable range for air velocity is 0 to 3 m/s.
- Metabolic rate. According to the activity status and ASHRAE Standard 55-2020^13^ metabolic rate comparison table, the metabolic rate range is 0.7 to 2.0 met.
- Clothing insulation. When clothing insulation is less than 0.02 clo, the body can be considered as being completely naked^41^, which will not expected to occur in field investigations. Based on existing research^28^ and the clothing insulation table in the standard^13^, there are no reported case of clothing insulation greater than 3.0 clo. Therefore, a reasonable range for clothing insulation is considered to be within 0.02 to 3.0 clo.
- Subjective evaluation. The range of TSV is set as -3 to 3, the range of TCV is set as 0 to 5, and the range of TAV is set as -1 to 1 based on the subjective evaluation scale used.

### Outlier detection

**Fig. 5:**
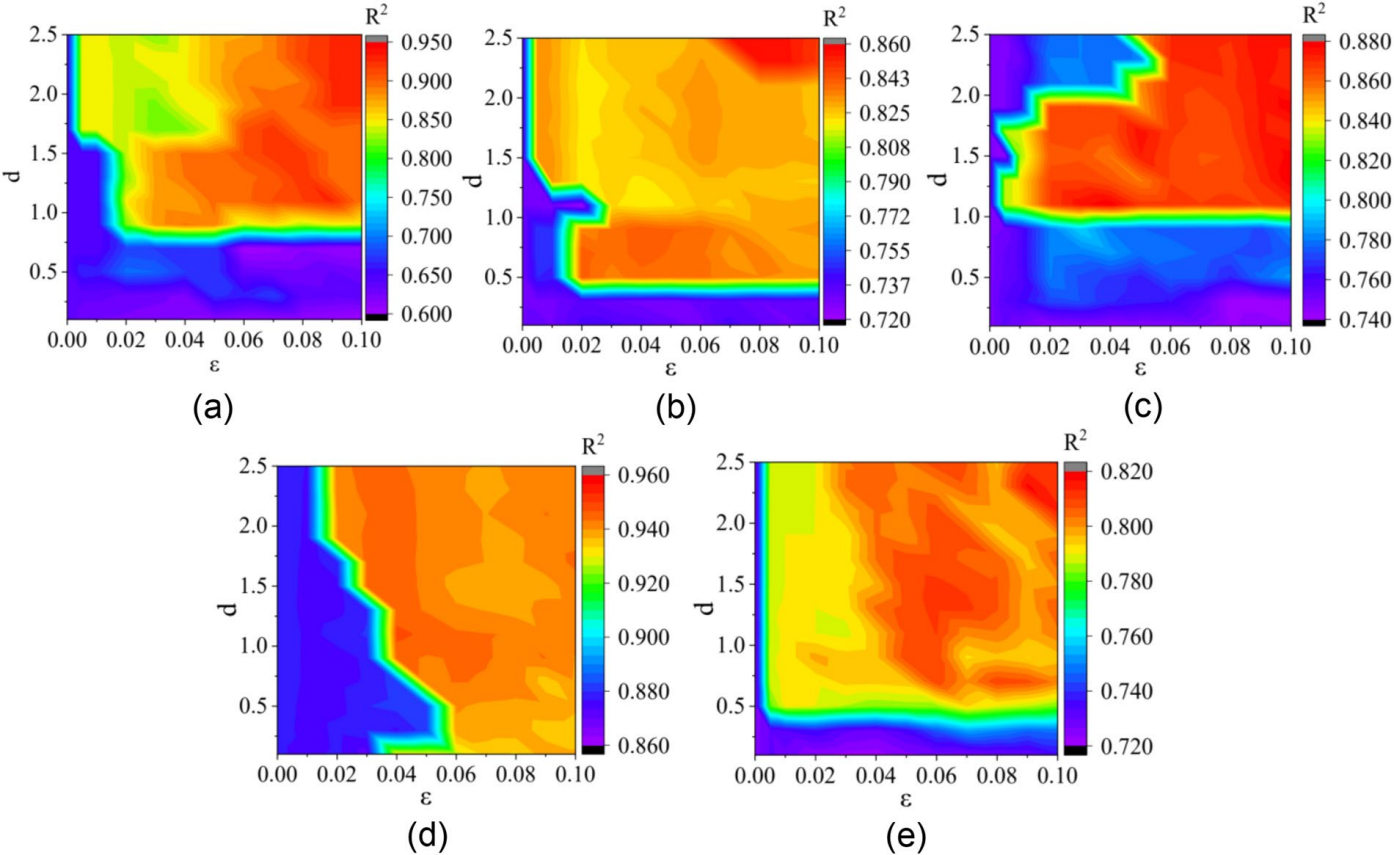
Contour diagram of parameter variation in different climate zones: (**a**) Severe cold zone; (**b**) Cold zone; (**c**) Hot summer & cold winter zone; (**d**) Hot summer & warm winter zone; (**e**) Mild zone.

During indoor data acquisition, a mismatch between the indoor thermal environment and the subjective evaluation may occur due to respondents’ misunderstanding of the questionnaire or lack of care in filling it out. To address this issue, outlier detection is performed to identify any data points significantly different from the rest of the data. Digital statistics and graphic visualization technology are used to visualize the distribution of each variable and detect outliers. However, visual observation and simple judgment cannot effectively detect outliers for TSV, so a two-step framework outlier detection method based on SET distance is used (Fig. 5). The principle of this method is to calculate the average distance between each sample point and its nearest K samples and compare the calculated distance (d) with a threshold value (ε). If the distance is larger than the threshold value, it is considered an anomaly^42^. This method has been shown to effectively distinguish outliers from inter-individual variabilities in thermal demand. For data not within the threshold range, we eliminate them and form the final dataset. Finally, the best parameter values for different climate zones are obtained: d value is 1.7, ε value is 0.005 in a severe cold zone; d value is 1.5, ε value is 0.005 in a cold zone; d value is 1.1, ε value is 0.005 in a hot summer & cold winter zone; d value is 2.3, ε value is 0.02 in a hot summer & warm winter zone; and d value is 0.7, ε value is 0.01 in a mild zone.

**Fig. 6:**
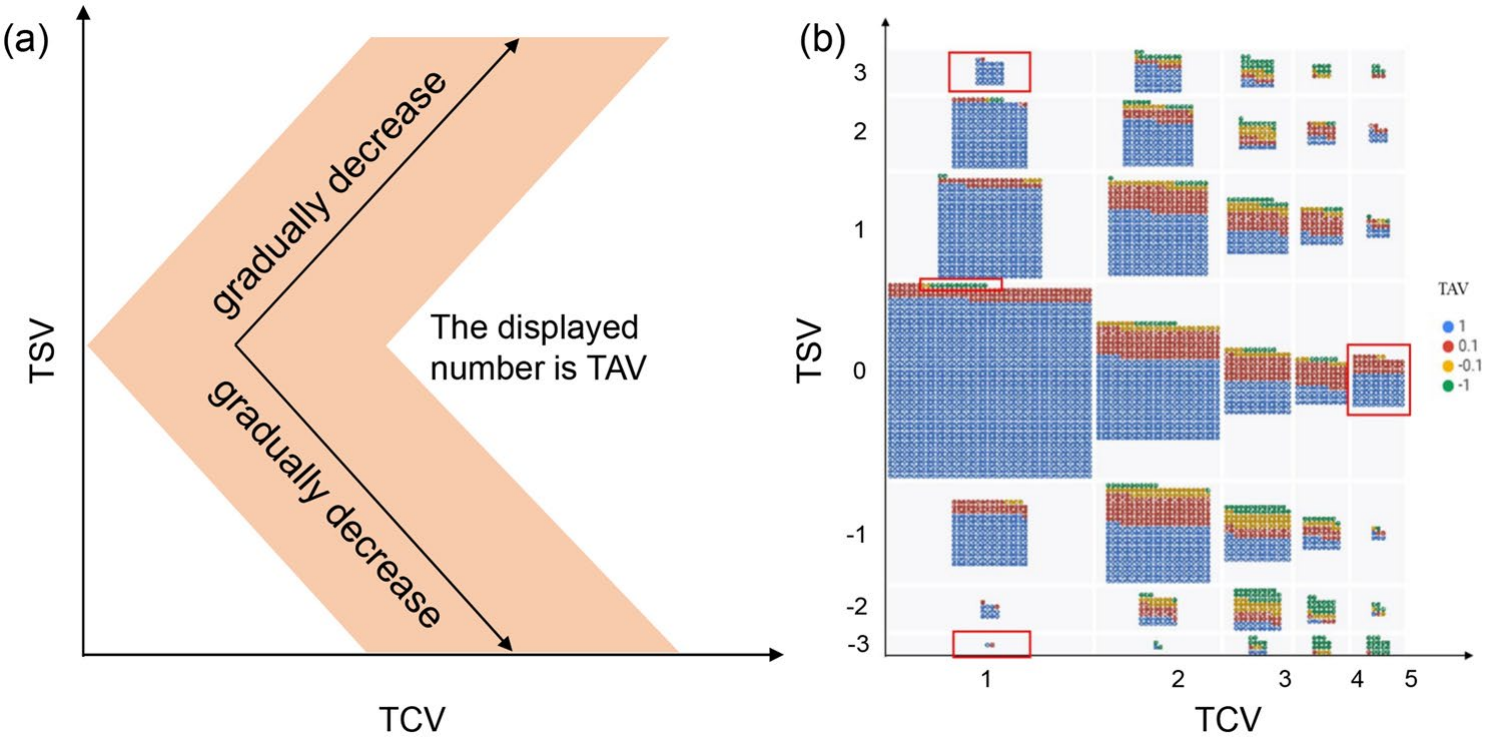
Theoretical (**a**) and actual (**b**) relationship among TSV, TAV and TCV.

To detect abnormal values in TCV and TAV, a visual method is employed based on the outlier detection of TSV. When TSV tends towards neutral, TCV tends to be comfortable and TAV tends to be acceptable. Conversely, when TSV tends towards cold or hot, TCV tends to be uncomfortable and TAV tends to be unacceptable. The TCV is taken as the x-axis, and the TSV as the y-axis, with data displayed as the TAV to obtain the theoretical relationship diagram between the three (Fig. 6a). By comparing the theoretical relationship diagram with the actual diagram, the three evaluation indexes can be considered outliers if they were contradictory (Fig. 6b).

**Fig. 7:**
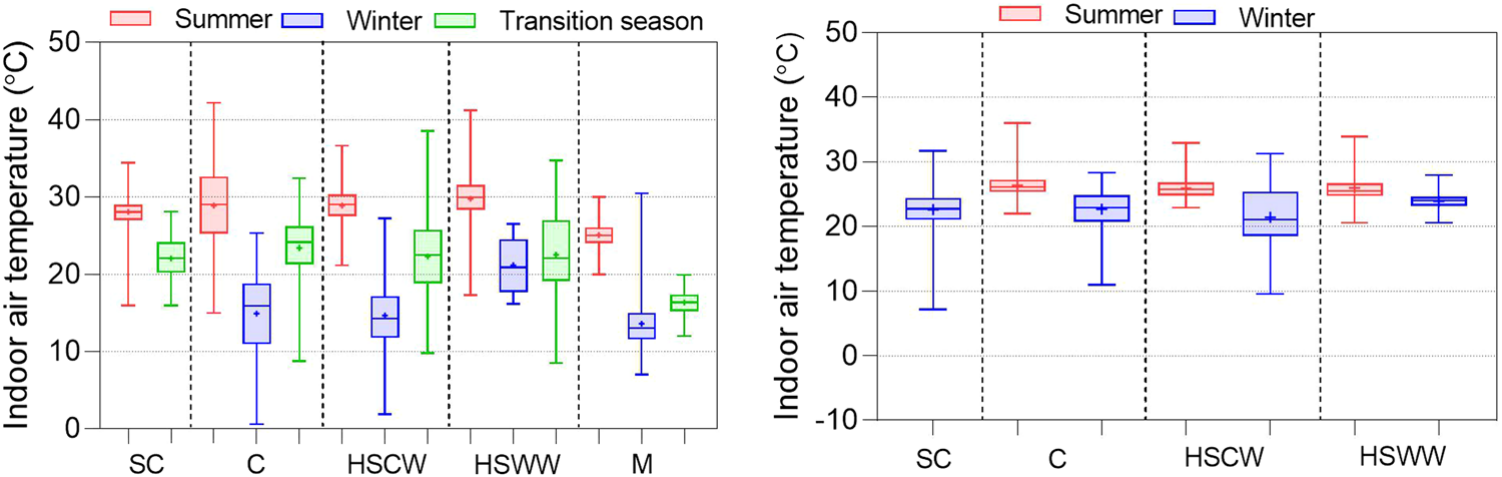
Indoor air temperature distributions in different climate zones, seasons, and operation modes.

**Fig. 8:**
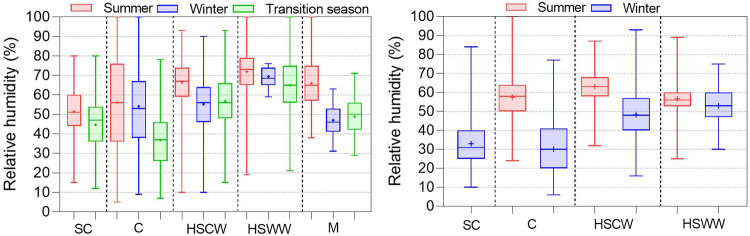
Indoor relative humidity distributions in different climate zones, seasons, and operation modes.

In this section, we present the distribution results of the physical environment parameters and subjective evaluations to ensure they conform to expected results and verify data quality. Figures 7, 8 displays the distribution of indoor air temperature and relative humidity in different climate zones, seasons, and operation modes. The indoor air temperature ranges from -5 to 45 °C, and the indoor relative humidity ranges from 5% to 100%. Under HVAC + Mixed mode operations, average indoor temperature in summer is significantly lower than that naturally ventilated buildings while generally higher in winter. This confirms that buildings with equipment involved in operation have a more comfortable indoor thermal environment than naturally ventilated conditions. Indoor relative humidity generally follows a seasonal pattern, with low values in winter and high values in summer, and the degree of variation differs in different climate zones.

**Fig. 9:**
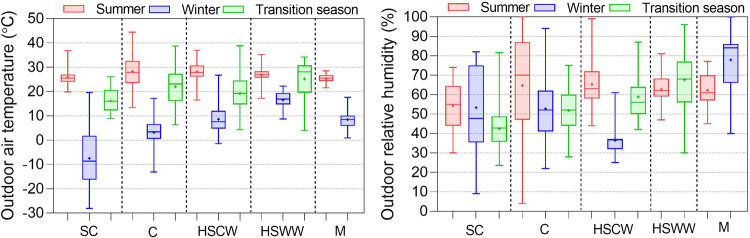
Outdoor air temperature and relative humidity distributions in different climate zones and seasons.

Outdoor environmental parameters are essential for developing thermal comfort prediction models and setting design parameters. Figure 9 presents the outdoor air temperature and relative humidity in various climate zones. The results indicate that average outdoor temperatures in summer and transition seasons across different climate zones are relatively similar, ranging mostly from 25 °C to 35 °C in summers and 15 °C to 25 °C in transition seasons. In winter, the average temperature varies widely in different regions.

**Fig. 10:**
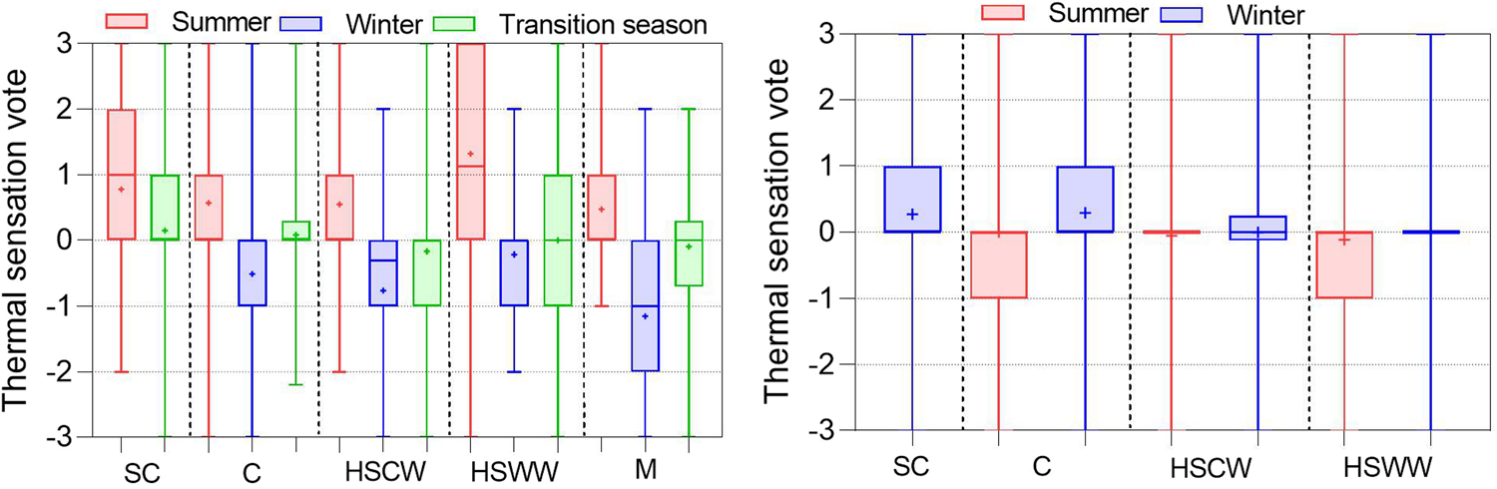
Thermal sensation vote in different climate zones, seasons, and operation modes.

Figure 10 shows the distribution of thermal sensation votes (TSV) in the dataset for different climate zones. We found there is a strong correlation between thermal sensation and indoor air temperature. Under the natural ventilation mode, the distribution of TSV is wide in all seasons due to the broad range of indoor air temperature distribution. The overall trend is that TSV is mainly distributed on the warm side (TSV > 0) in summer, on the cold side (TSV <0) in winter, and near neutral (TSV = 0) in the transitional season. Under the HVAC + Mixed mode operations, the difference of TSV in different climate zones is not significant, and most of the TSV in winter and summer are distributed in the range of [-1, 1]. The mean value of TSV in winter is located on the warm side (TSV > 0) because of the high heating temperature in the cold and severe cold zones.

**Fig. 11:**
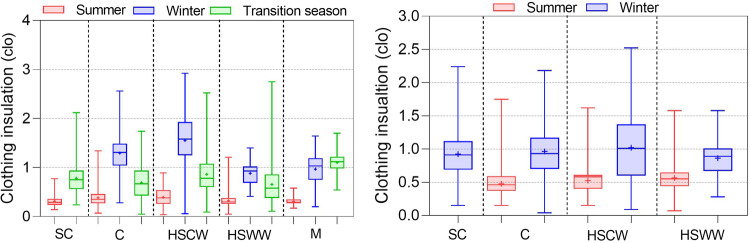
Clothing insulation in different climate zones, seasons, and operation modes.

Clothing insulation is a crucial factor affecting human thermal comfort^28^. In the data consistency check, we specified a reasonable range of clothing insulation is 0.02 to 3.0 clo. Figure 11 illustrates the distribution of clothing insulation, which is significantly influenced by outdoor climate. As anticipated, there is a general trend of higher insulation in winter than in transition seasons and summer. Differences in the distribution of clothing insulation can also be observed.

### Conclusion and next steps

The Chinese thermal comfort dataset consists of 41,977 data sets covering five distinct climate zones (severe cold zone, cold zone, hot summer & cold winter zone, hot summer & warm winter zone, and mild zone), four building types (office, residential, classrooms, and dormitory), and three operation modes (naturally ventilation, HVAC, and Mixed mode). Following quality control and outlier elimination, the dataset was utilized to develop Chinese adaptive models and formulate corresponding standards. However, some study data is not included in this dataset, and we will continue to expand the dataset and make it publicly available, so that the thermal comfort society can benefit from the work. The dataset can be used to model human thermal comfort, adaptive behavior, and investigate regional differences in indoor thermal conditions and occupants’ thermal comfort responses. Furthermore, it will be added to the ASHRAE database II, expanding its accessibility and usefulness.

## Usage Notes

The dataset has been uploaded to a public domain of the Open Science Framework website, users can download data by referring to Reference [34] (10.17605/OSF.IO/D465N). In addition, we also upload the data to the website^43^ http://buildingdata.xauat.edu.cn/TC-dataset/. Users can query and download the required data from the dataset according to different selection criteria. There are three different levels of data: Chinese Thermal Comfort Dataset Class I, Chinese Thermal Comfort Dataset Class II, and Chinese Thermal Comfort Dataset Class III.

## Data Availability

All the codes used to clean the raw datasets have been uploaded to publicly available on the OSF data repository^34^ (10.17605/OSF.IO/D465N). The data can be analyzed using software such as R or MATLAB.
